# An approach to integrate population mobility patterns and sociocultural factors in communicable disease preparedness and response

**Published:** 2021

**Authors:** Rebecca D. Merrill, Ali Imorou Bah Chabi, Elvira McIntyre, Jules Venance Kouassi, Martial Monney Alleby, Corrine Codja, Ouyi Tante, Godjedo Togbemabou Primous Martial, Idriss Kone, Sarah Ward, Tamekloe Tsidi Agbeko, Clement Glèlè Kakaı

**Affiliations:** 1 US Centers for Disease Control and Prevention, Atlanta, USA.; 2 Abidjan Lagos Corridor Organization, Benin, Nigeria.; 3 Perspecta and US Centers for Disease Control and Prevention, Atlanta, USA.; 4 Ministry of Health, Lomé, Togo.; 5 Ministry of Health, Benin, Nigeria.

## Abstract

Complex human movement patterns driven by a range of economic, health, social, and environmental factors influence communicable disease spread. Further, cross-border movement impacts disparate public health systems of neighboring countries, making an effective response to disease importation or exportation more challenging. Despite the array of quantitative techniques and social science approaches available to analyze movement patterns, there continues to be a dearth of methods within the applied public health setting to gather and use information about community-level mobility dynamics. Population Connectivity Across Borders (PopCAB) is a rapidly-deployable toolkit to characterize multisectoral movement patterns through community engagement using focus group discussions or key informant interviews, each with participatory mapping, and apply the results to tailor preparedness and response strategies. The Togo and Benin Ministries of Health (MOH), in collaboration with the Abidjan Lagos Corridor Organization and the US Centers for Disease Control and Prevention, adapted and applied PopCAB to inform cross-border preparedness and response strategies for multinational Lassa fever outbreaks. Initially, the team implemented binational, national-level PopCAB activities in March 2017, highlighting details about a circular migration pathway across northern Togo, Benin, and Nigeria. After applying those results to respond to a cross-border Lassa fever outbreak in February 2018, the team designed an expanded PopCAB initiative in April 2018. In eight days, they trained 54 MOH staff who implemented 21 PopCAB focus group discussions in 14 cities with 224 community-level participants representing six stakeholder groups. Using the newly-identified 167 points of interest and 176 routes associated with a circular migration pathway across Togo, Benin, and Nigeria, the Togo and Benin MOH refined their cross-border information sharing and collaboration processes for Lassa fever and other communicable diseases, selected health facilities with increased community connectivity for enhanced training, and identified techniques to better integrate traditional healers in surveillance and community education strategies. They also integrated the final toolkit in national- and district-level public health preparedness plans. Integrating PopCAB in public health practice to better understand and accommodate population movement patterns can help countries mitigate the international spread of disease in support of improved global health security and International Health Regulations requirements.

## Introduction

Human movement influences the geospatial spread of communicable disease. Several factors impact the complex spatial and temporal patterns of that movement, including health status, travel distance, duration, and purpose, the built environment, economic systems, and homophily (McPherson, Smith-Lovin, and Cook, 2001; Castelli, 2018). This social, economic, and cultural connectivity and associated human mobility challenges public health systems’ capacity to detect and respond to communicable disease events efficiently. These challenges increase when movement crosses an international border and disparate public health and medical systems, with unique preparedness, response, and risk assessment capacities, are impacted (Wangdi et al., 2015; Moore et al., 2018; Holmes et al., 2016).

Contact tracing and travel history investigations are proven strategies to tailor responsive interventions immediately following a communicable disease event (Armbruster and Brandeau, 2007; Hellewell et al., 2020). These methods facilitate gathering data about people and places with an elevated risk of recent exposure to enable resource allocation for rapid identification and isolation of ill individuals. While critical to response efforts, these methods have some limitations. Public health practitioners must gather individual-level information through participant recall or analyses of extant data to illuminate recent travel and social interactions and must exhaustively visit and track the identified contacts for a defined amount of time. Significantly, these methods do not directly contribute to a broader assessment of where disease transmission could occur if not all contacts or visited locations were identified, found, or followed over time.

Quantitative analytic methods using call data records, mobile phone location history data, mathematical modeling, periodic census and population-based demographic surveys, chronological satellite imagery, and phylogenetic analyses can explore spatio-temporal disease transmission dynamics accounting for population mobility (Mazzoli et al., 2020; Bharti et al., 2016; Aleta et al., 2020; Dudas et al., 2017; Ruktanonchai et al., 2018; Riascos and Mateos 2017; Kraemer et al., 2020). Each method achieves unique and complementary goals towards quantifying the risk of exposure to, importing, and exporting communicable disease threats at geographic locations, among population groups, or along travel routes. These techniques continue to be critical for increasing the efficiency and effectiveness of public health programming to mitigate the spread of infectious disease and the impact of humanitarian crises. However, these methods do not address the ‘who’ and ‘why’ of movement patterns and social interactions that influence disease spread.

Social network analysis is a method to investigate and describe the system of dependency between and influence of individual and group relationships (Tabassum et al., 2018). This analytic approach is often applied when studying formal and informal population movements for various geographic scales and sectors (Akbari, 2021; Valerio et al., 2020). Social network analyses have also contributed to better understanding the relative influence of social and spatial factors on infectious disease spread (Giebultowicz et al., 2011). The richer understanding this analytic method generates about transmission dynamics contributes to adapting public health programs to unique multisectoral contexts. Nonetheless, there remains a gap between conducting social network analyses and decision-making in applied public health settings (Rothenberg and Costenbader 2011).

Public health stakeholders, including ministries of health, responding to communicable disease events may lack the methodological resources or time to implement the described risk forecasting and social network analyses. To complement the sophisticated approaches, response leadership could benefit from access to methods to characterize general sociocultural and population movement patterns that may impact disease spread.

In January 2016 the Benin Ministry of Health (MOH) reported its second-ever outbreak of Lassa fever, a hemorrhagic fever spread to humans through the “multimammate rat” (*Mastomys natalensis*) reservoir with a case fatality ratio ≥15% among hospitalized patients (WHO 2016; Kofman, Choi, and Rollin, 2019). Three weeks later, the Togo MOH reported their first-ever Lassa fever case (Patassi et al., 2017). Recognizing that Lassa fever is endemic in Nigeria and that community connectivity and population mobility is high across the region, the Togo and Benin MOH sought to understand better the risk of regional spread (Kakaī et al., 2020). We describe how the Togo and Benin MOH implemented the flexible, rapidly-deployable Population Connectivity Across Borders (PopCAB) toolkit to characterize community connectivity dynamics and travel patterns at local and national levels. We also explore how the MOH applied the results to enhance national and cross-border Lassa fever outbreak preparedness and response strategies that accommodated unique, informal migration patterns and health care seeking behaviors along the routes.

## Population connectivity across borders (PopCAB) method

The overall goal of PopCAB is to facilitate better integrating population mobility in public health surveillance, programming, preparedness, and response initiatives. The method includes qualitative, spatial, and quantitative data collection techniques to characterize and interpret the who, where, when, why, and how of human mobility and community connectivity. Stakeholders can rapidly adapt and implement PopCAB within a matter of hours to support an outbreak response or over extended periods to inform longer-term public health initiatives.

**Fig. 1:**
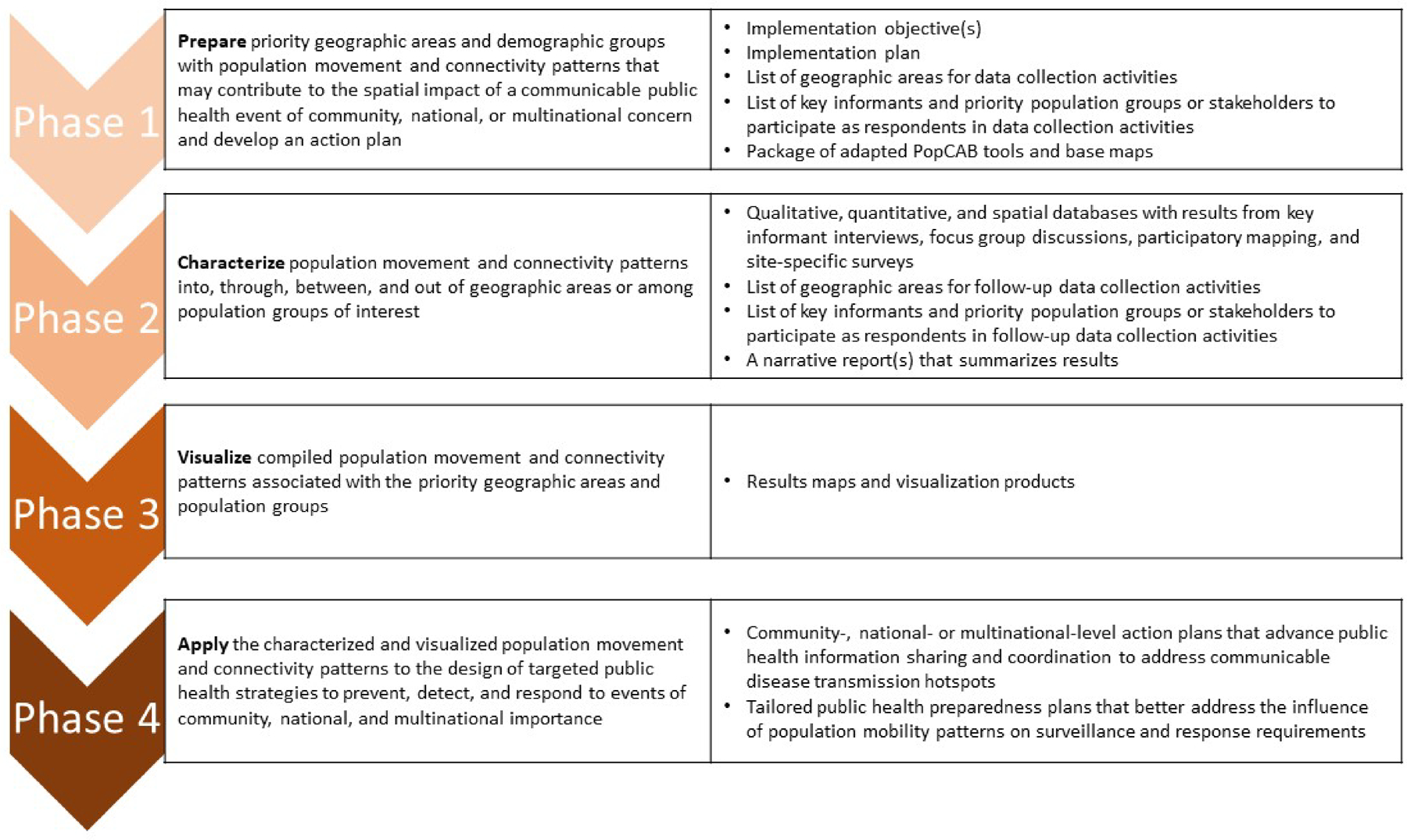
List of the four phases of Population Connectivity Across Borders (PopCAB) implementation. The first column contains information about the goal of each phase and the second columns includes a list of associated outputs.

PopCAB is a mixed-methods approach involving four implementation phases: prepare, characterize, visualize, and apply (Fig. 1). Before initiating Phase 1, interested stakeholders identify the PopCAB implementation “team” composed of (a) leadership to manage the activities, finalize the objectives and adapted toolkit, and ensure the products address the defined objectives, (b) individuals to implement the field activities, (c) a data manager, and (d) a Geographic Information System (GIS) analyst to use, create, and manage maps and spatial data. Team members can serve in more than one role. Together, they invite traditional and non-traditional public health stakeholders to participate in key informant interviews (KIIs) and focus group discussions (FGDs). Both the KIIs and FGDs include participatory mapping to document spatial aspects of movement and connectivity. The team can also conduct quantitative, individual-level surveys at specific points of interest to gather additional, relevant data about movement patterns.

### Phase 1: Prepare.

The purpose of Phase 1 is to ensure the team is prepared to implement targeted PopCAB activities. Notably, the team, in collaboration with relevant stakeholders, defines the context-specific PopCAB objective(s). These objectives can be localized in time and space to, for example, characterize current movement patterns during an outbreak among women and children associated with a specific market or can be more expansive to illuminate regional, annual, informal migration patterns to inform more efficient multinational public health preparedness strategies.

With the objective(s) defined, the team reviews existing information to ensure the activities in subsequent Phases leverage extant knowledge. Based on the objectives and consolidated information, the team then:

- develops a work plan with a timeline and assigned responsibilities to complete the 4-phase cycle,
- lists key informants and stakeholder groups to invite to participate in KIIs and FGDs in Phase 2 activities,
- identifies priority geographic areas for Phase 2 activities,
- adapts the generic toolkit materials to the unique environmental, political, and public health context, and
- designs base maps for the participatory mapping activities.

The base maps should cover the priority geographic area(s) for which information will be gathered and be designed at the same scale as the data that will be collected. For example, if an objective is to gather data on general population mobility across a multi-country region, the base maps should reflect all the selected countries. Alternatively, if the objective is to gather data on population mobility within a specific community in a sub-national administrative area, more detailed community-level maps are appropriate. It is important to include sufficient reference information to enable participants to orient themselves to the map including villages or towns, parks, roads, political boundaries, rivers, and lakes. Additional details can include health facilities, large markets, or points of entry. However, base maps should not include excess information, such as names of every road or all markets, which may overwhelm the participants or hinder their ability to annotate the map during the KII or FGD. When resources allow, 36 × 48 inch printed base maps are ideal for the participatory mapping component. They provide printed space for sufficient detail and are big enough to allow multiple participants to gather around the map comfortably. When the team cannot print large maps, the team can print the larger map on multiple pieces of paper and tape them together. Alternatively, the GIS analyst can make smaller maps, such as 8.5 × 11 inch or 11 × 17 inch sizes.

The outputs of Phase 1 include well-defined PopCAB objective (s) and implementation plan, a list of geographic areas in which to conduct data collection activities, a list of key informants and priority population groups to invite to participate as respondents in data collection activities, and a package of PopCAB tools and base maps adapted to the specific opportunity.

### Phase 2: Characterize.

The purpose of Phase 2 is to gather information on population movement and connectivity through community engagement, empowering the stakeholders to understand and contribute to the process of incorporating mobility in public health initiatives.

#### Key informant interviews and focus group discussions with participatory mapping.

The team creates one or more field team(s) to facilitate data collection activities with key informants and stakeholders identified in Phase 1. Each field team must have at minimum three members: a discussion facilitator and two note takers. In short, a facilitator guides one participant (KII) or six to 10 participants (FGD) through an open-ended discussion using an adapted PopCAB discussion guide (Onwuegbuzie et al., 2009). To create the adapted guide, the team tailors the questions in the template tool to ensure the discussions inform the objective(s) and respond to a stakeholder-specific context (Table 1). For an FGD, the facilitator assigns all participants a unique, numerical identifier. Throughout the discussion, two note takers record, verbatim, the facilitator’s questions and all participants’ comments, including the unique identifier for the participant who stated the information, along with additional comments about the interactions between those individuals and between the facilitator and participants. With appropriate participant consent, the team can also audio record the discussion to use as a resource when quality checking the recorded, verbatim notes.

The team chooses a location to implement the KIIs or FGDs where participants are comfortable and have sufficient space to complete participatory mapping. The PopCAB participatory mapping method advances classic, hand-drawn community mapping methods by integrating printed, spatially-accurate base maps and having the facilitator, or a selected participant, annotate points of interest, migration pathways, geographic areas of interest, and other spatial information directly on the map. The facilitator assigns all annotations an unique alphabetical identifier to aid in linking information recorded on the map with the verbatim discussion notes. For example, suppose a participant with a unique identifier 6 described visiting a specific market assigned a “B” when annotated on the map. In that case, the discussion notes should document “Participant 6: I visit the market (B) on Sunday.”

The facilitator ensures active engagement, even for those who have never seen a printed map or who are illiterate, by first providing a dedicated orientation to cardinal directions and a few relevant, marked locations or natural features on the base map. The facilitator then asks the participants to point out relevant features such as an important lake, market, or health facility. For each discussion, the facilitator uses a new base map free of annotations from any other discussion. Where a printed map is not available, a hand-drawn map remains a viable option to document spatial information discussed during a PopCAB event.

After each KII and FGD event the facilitator and note takers review the written notes and annotated map(s) to ensure they thoroughly and accurately documented the discussion and participatory mapping activities. Specifically, the recorded notes must document the facilitator’s questions, the participants’ comments by participant unique identifier, and reflections on interactions between the facilitator and participants and among the participants. In addition, the field team must ensure they assign and link unique identifiers for spatial information in the written notes and on the annotated maps.

#### Site-specific, individual-level survey.

The team identifies one or more survey team(s), composed of at least two interviewers each, to facilitate individual-level surveys at priority sites, such as markets, health facilities, or central bus stops, to gather quantitative information about location-specific population movement patterns including modes of transport, travel purpose, travel duration, travel origin, and where interviewees may travel next. The team identifies relevant sites using results from the KIIs and FGDs. These sites are selected to address project objectives such as attracting visitors from a population group of interest, being highly visited during a priority time of year, or involving movement pathways near a specific health facility. After considering each site’s unique context, the team identifies specific times to conduct the structured interviews to capture site-specific periodicity in population movement. For example, for a market, the team may select a three-hour period the morning of an open market day and a morning when the market is closed in order to compare traveler trends on busy and quiet days.

The field team completes data collection using the survey questionnaire and sampling strategy adapted during Phase 1. The sampling strategy depends on the project objectives. The field team could interview only those who do not reside in the proximate community to learn about travel that originates in neighboring administrative units, or they could interview anyone who visits the site to learn about movement within and around the surrounding community. Absent a rigorous sampling strategy, the quantitative data will not represent a more generalized community or social network. However, the information can help characterize the potential magnitude of movement associated with key locations and associated geographic spread of communicable disease.

#### Data management and use.

The team completes qualitative data analysis using the KIIs and FGD discussion notes as described in more detail elsewhere (Onwuegbuzie et al., 2009). One unique overarching goal of the data management process is to create a database listing all mentioned locations and migration routes (location database). To create a spatial database, the GIS analyst uses the location database and the annotated map to enter coordinates by referencing information on the annotated paper maps and existing spatial datasets. When the team can visit identified points of interest, they can also use mobile data collection tools to gather location coordinates. By integrating a spatially accurate participatory mapping process, the GIS analyst can more rapidly transfer into a spatial database the Global Positioning System (GPS) coordinates for locations annotated on the map.

Using the compiled qualitative, spatial, and quantitative data, the team creates databases using structures outlined during Phase 1, completes analyses to address the objectives, and develops reports for dissemination. The implementation team also determines if they should complete additional PopCAB data collection events with other stakeholder groups or in other geographic areas. This Phases’ outputs include qualitative, quantitative, and spatial databases that capture information from KIIs, FGDs, participatory mapping, and site-specific surveys, a list of priority geographic areas and population groups for follow-up, and a narrative report(s) that summarizes results to inform the objectives.

### Phase 3: Visualize.

During this phase, the team creates static or interactive maps and visualization products that convey community connectivity across prioritized points of interest and geographic areas. Effectively visualizing the gathered information is critical to persuasively conveying and efficiently encouraging the translation of Phase 2 results for public health action, especially into actions to address PopCAB objectives defined in Phase 1. Importantly, these maps incorporate layers of information to address contextual, multisectoral considerations to respond to the stated PopCAB objectives and the evolving context.

The GIS analyst structures the gathered data with markers representing the points of interest and lines representing migration routes to create the results maps. Using the qualitative and quantitative databases, the team characterizes the points of interest and migration routes according to themes, including the built environment, cultural identities and behaviors, health care seeking behavior, or economic systems. The result maps can emphasize additional factors by symbolizing stakeholders, including seasonality, or incorporating a brief narrative about priority routes.

### Phase 4: Apply.

This phase involves translating Phase 2 and 3 outputs into tailored recommendations for designing public health interventions or action plans to incorporate community connectivity and population mobility better. In a preparedness context, these tailored plans could incorporate refresher training for community health volunteers on ill person surveillance and reporting one month before a forecasted increase in travelers migrating for annual agricultural work to a neighboring community. During an outbreak response, the action plans could identify strategies to allocate human and financial resources more effectively to strengthen surveillance capacity in locations or general areas forecasted to have an increased risk of disease translocation. At this point, the team can revisit the decision to implement additional PopCAB data collection events to expand the depth or breadth of information.

Phase 4 outputs include action plans that better incorporate risk assessments and forecasting results that address community connectivity and population mobility. These action plans can include adapted outbreak response strategies that target secondary communities with cultural connectivity to the outbreak zone; tailored public health preparedness plans that better address the influence of population mobility patterns on surveillance and response requirements; or community-, national-, or multinational-level action plans that advance public health information sharing and coordination to address communicable disease transmission hotspots.

## Application of PopCAB in Togo and Benin

### Round 1, Phase 1.

In early 2017, in collaboration with the US Centers for Disease Control and Prevention (CDC) and the Abidjan Lagos Corridor Organization (ALCO), the Togo and Benin MOH identified an overall need to strengthen cross-border public health surveillance and collaboration through an enhanced understanding of cross-border community connectivity. This need was immediately applicable due to an ongoing cross-border Lassa fever response to a series of cases following an index case identified by the Benin MOH on February 11 (WHO, 2017). The collaborators identified a PopCAB team, composed of leadership from the MOH Departments of Disease Surveillance, including the International Health Regulations National Focal Points, project leaders, and field staff from ALCO, and an epidemiologist, health scientist, and GIS analyst from CDC. The team clarified an initial objective to identify priority geographic areas for enhanced PopCAB fieldwork. To achieve this objective, the team listed critical stakeholders for a national-level PopCAB event that would represent health and non-health sectors with responsibilities relating to border health and security systems or health care seeking among mobile populations: public health leadership, including MOH representatives, police officials, immigration officials, and mayors, environmental health specialists, and medical doctors, especially those who work in border areas. The team integrated the first PopCAB event in a pre-planned, multisectoral binational meeting in March 2017.

**Fig. 2:**
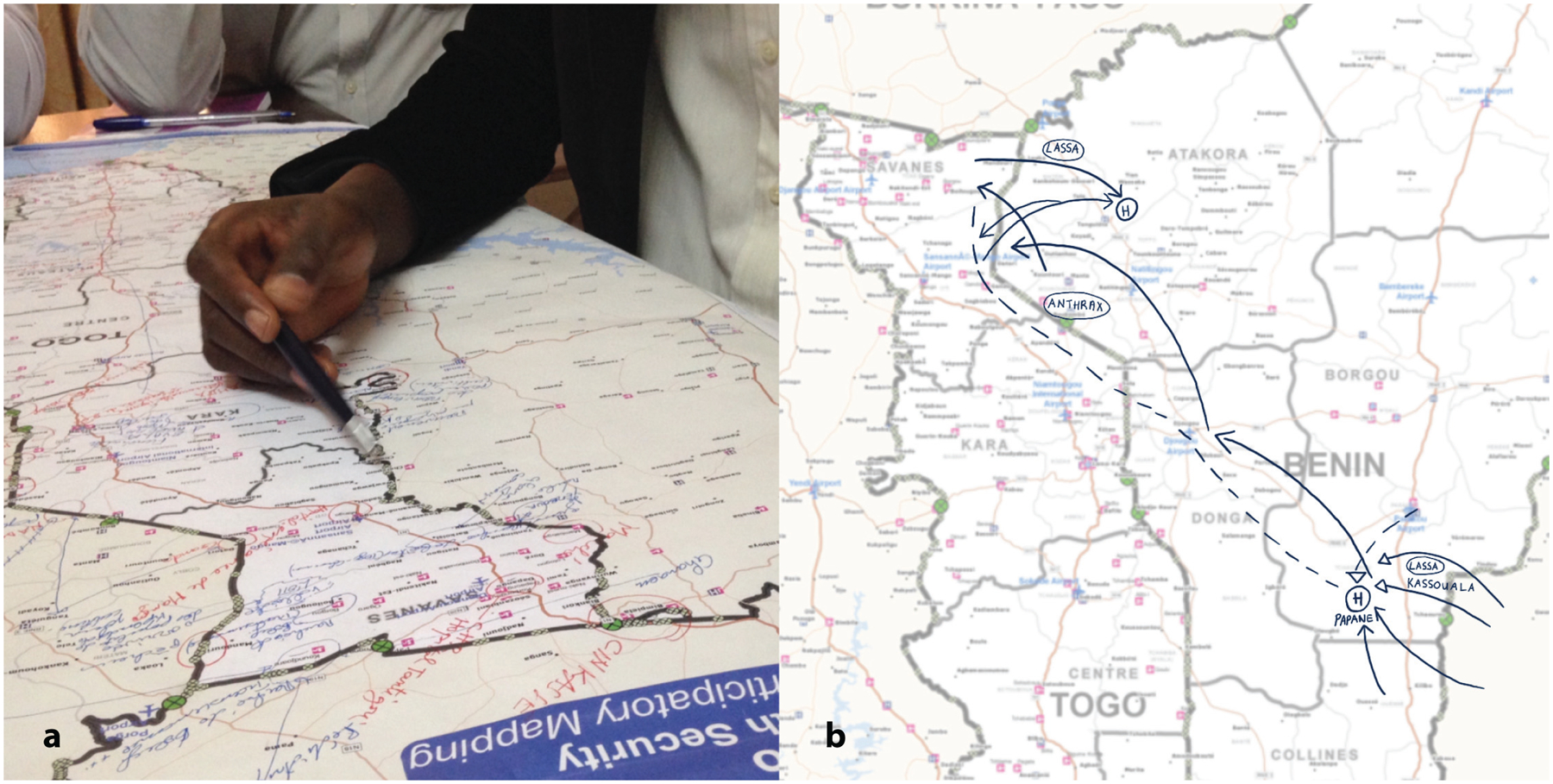
Annotated maps from Population Connectivity Across Borders (PopCAB) participatory mapping completed during focus group discussions at a binational meeting between Togo and Benin. **a** Photo of participants of the Togo focus group discussion annotating a national-level map; **b** Digital enhancement of annotations on the Benin national map.

The team consolidated existing information, including spatial data, on seasonal migration between Togo, Benin, and Nigeria and previous Lassa fever outbreaks in the region. The GIS analyst created multiple base maps scaled to the national level for Togo and Benin (Fig. 2). These base maps showed roads, parks, administrative boundaries, rivers and lakes, major health facilities, and points of entry. The team also adapted PopCAB toolkit materials to the context by translating the materials into French and adding probing questions to reflect movement and connectivity considerations relevant to the participating stakeholders.

All described PopCAB activities in Togo and Benin were designed to inform public health practice rather than contribute to generalizable knowledge. CDC’s role in providing technical support to the collaboration was approved through formal review determining that the project does not meet the definition of research. All FGD participants agreed to willingly participate after hearing an orientation script about the activity’s purpose.

### Round 1, Phase 2.

During the meeting, participants dedicated one hour to complete the country-level PopCAB FGDs with participatory mapping and one hour to discuss the results as a combined, binational group (Fig. 2). All participants consented to involvement. They compared national, annotated maps to identify patterns and discussed strategies to apply the results. After the meeting the team created spatial and qualitative datasets using the annotated maps and discussion notes.

### Round 1, Phase 3.

**Fig. 3:**
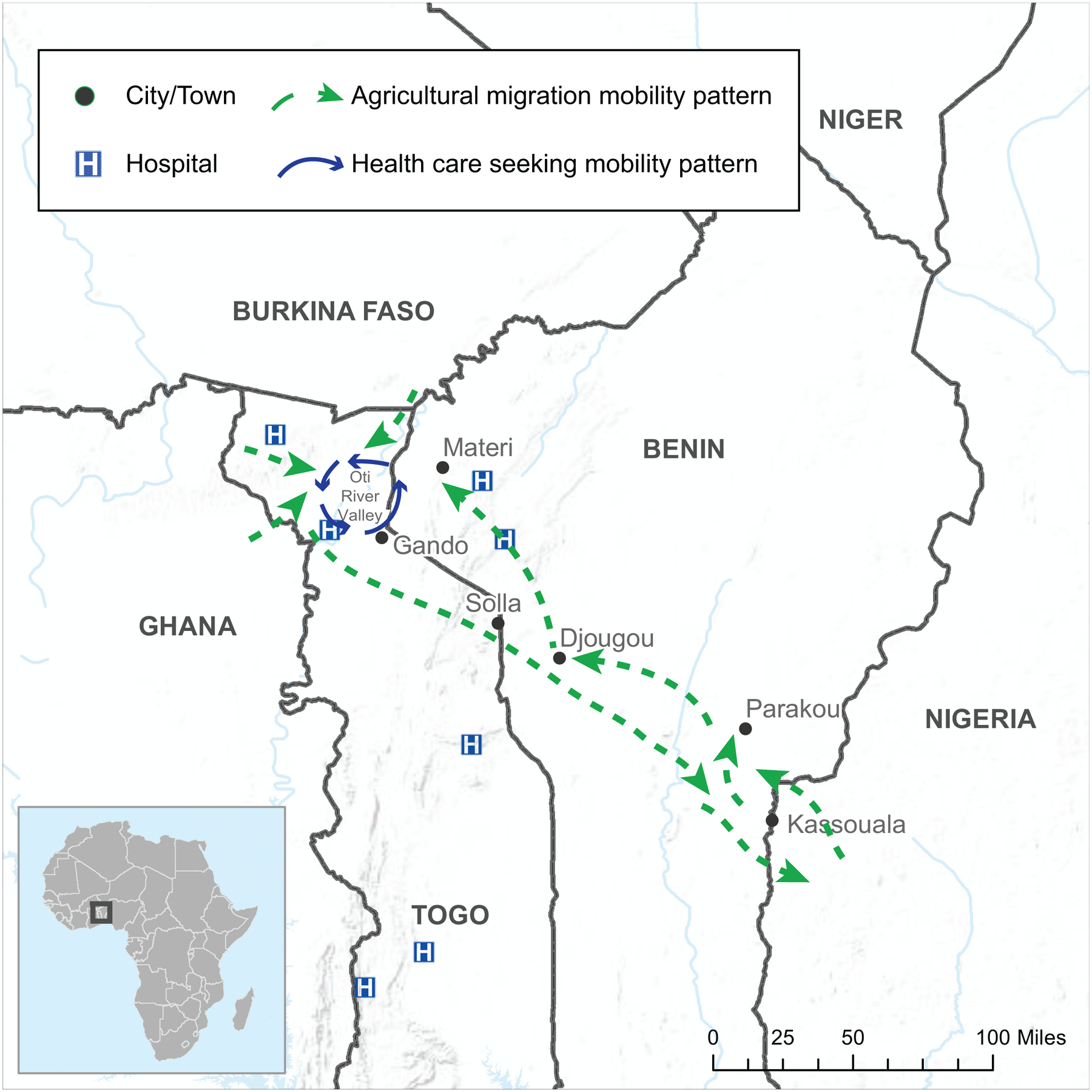
Example results map from Round 1 Population Connectivity Across Borders (PopCAB) implementation, highlighting two thematic drivers of cross-border mobility patterns. This map reflects results from two focus group discussions, one each with participants from Togo and Benin.

The team created digital maps to visualize the wealth of gathered data further. They incorporated qualitative data from the FGD notes reflecting multisectoral comments about the movement and identified locations. In addition to seeing the overlap of numerous movement pathways, users could filter data shown on these national and binational map products by reasons for the movement, e.g., health care seeking, economic, agricultural (Fig. 3).

### Round 1, Phase 4.

During the binational meeting in March 2017, public health leadership identified geographic areas with increased cross-border connectivity for enhanced binational collaboration and health facilities along migration pathways for additional training to identify and respond to illness among travelers. After reviewing in more detail the qualitative notes and maps showing migration pathways and Lassa fever outbreak data, the team identified the need to implement PopCAB in northern Benin and Togo where there was a distinct relationship between a seasonal, circular migration pathway between Togo, Benin, and Nigeria with geographically overlapping cross-border health care seeking patterns (Fig. 3). The MOH representatives on the team highlighted travel history information in the recent outbreak that suggested an association between this seasonal migration pathway and cross-border Lassa fever importation.

### Round 2, Phase 1.

The team defined objectives for the second round of PopCAB to rapidly characterize a) population movement patterns associated with cross-border, circular migration between northern Togo, Benin, and Nigeria and b) traditional and formal health care seeking behaviors along the general route. They prepared to implement this round in April 2018. The team decided not to incorporate site-specific surveys because the need for quantitative information was minimal.

Using additional information gathered from published reports, national public health data, and multisectoral discussions, they identified key national- and district-level stakeholders to invite as participants in Phase 2 field activities. More specifically, they selected stakeholders who could provide information about cross-border migration patterns and health care seeking behaviors along those routes: public health surveillance officers, border officials, migrants, health care professionals, traditional healers, and transporters. They also identified priority geographic areas including 10 urban centers across the northern regions of Kara and Savanes Regions in Togo and Atakora, Donga, and Borgou Departments in Benin. For each geographic area, the team identified national-level and district-level MOH staff to compose nine field teams (four in Togo and five in Benin), each with one team manager, one facilitator, and two note takers. The GIS analyst used previous base maps scaled to Togo and Benin’s national level and developed one map scaled to each of the pre-selected sub-districts within the Regions and Departments. The team further adapted the FGD facilitation guides by incorporating qualitative question elements to address seasonal and cross-border migration and traditional and formal health care seeking behaviors when migrating.

### Round 2, Phase 2.

**Fig. 4:**
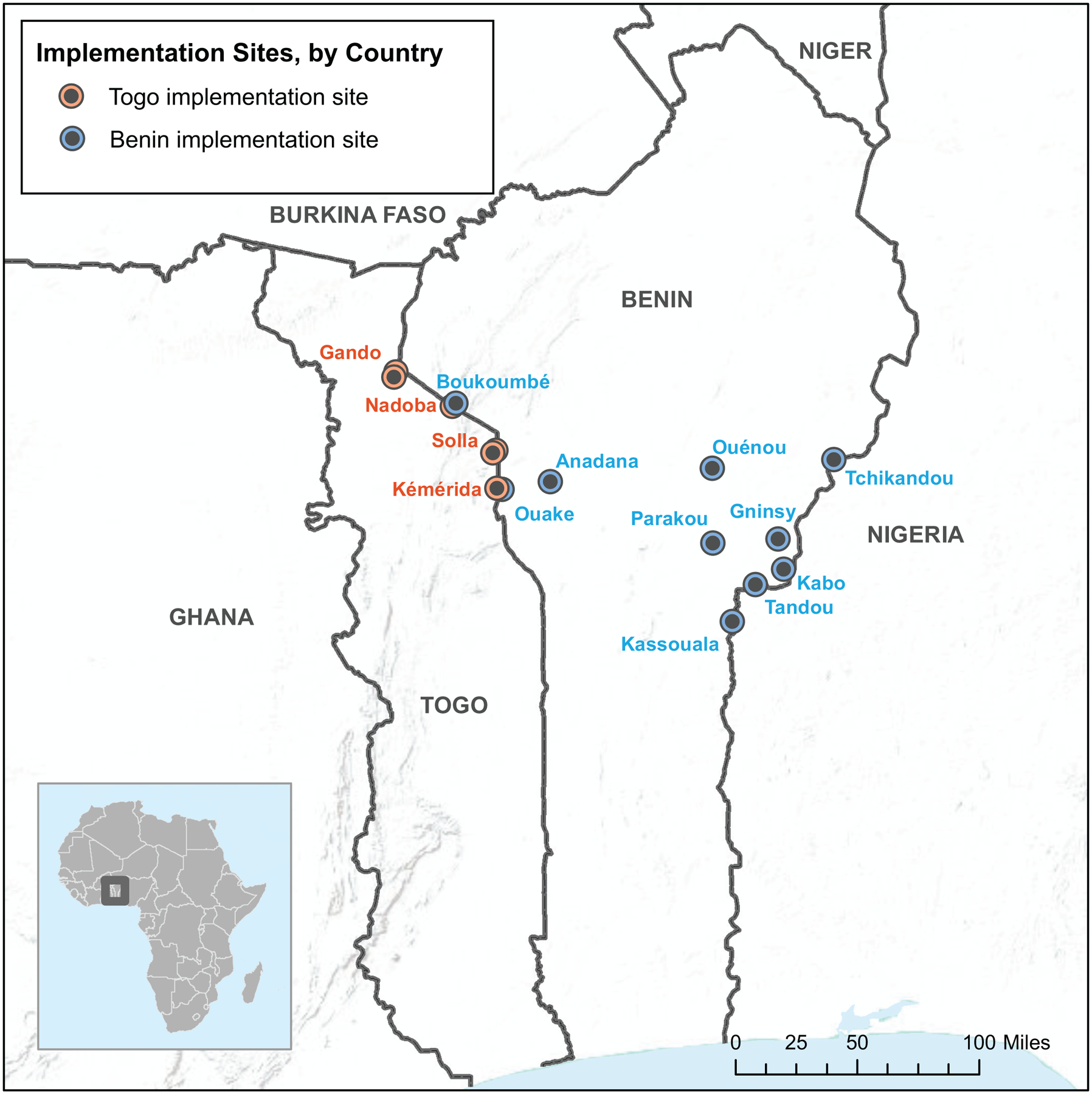
Round 2 Population Connectivity Across Borders (PopCAB) implementation sites in Togo and Benin, April 2018. The binational map of Togo and Benin highlights the 14 cities where the field teams implemented PopCAB events.

**Fig. 5:**
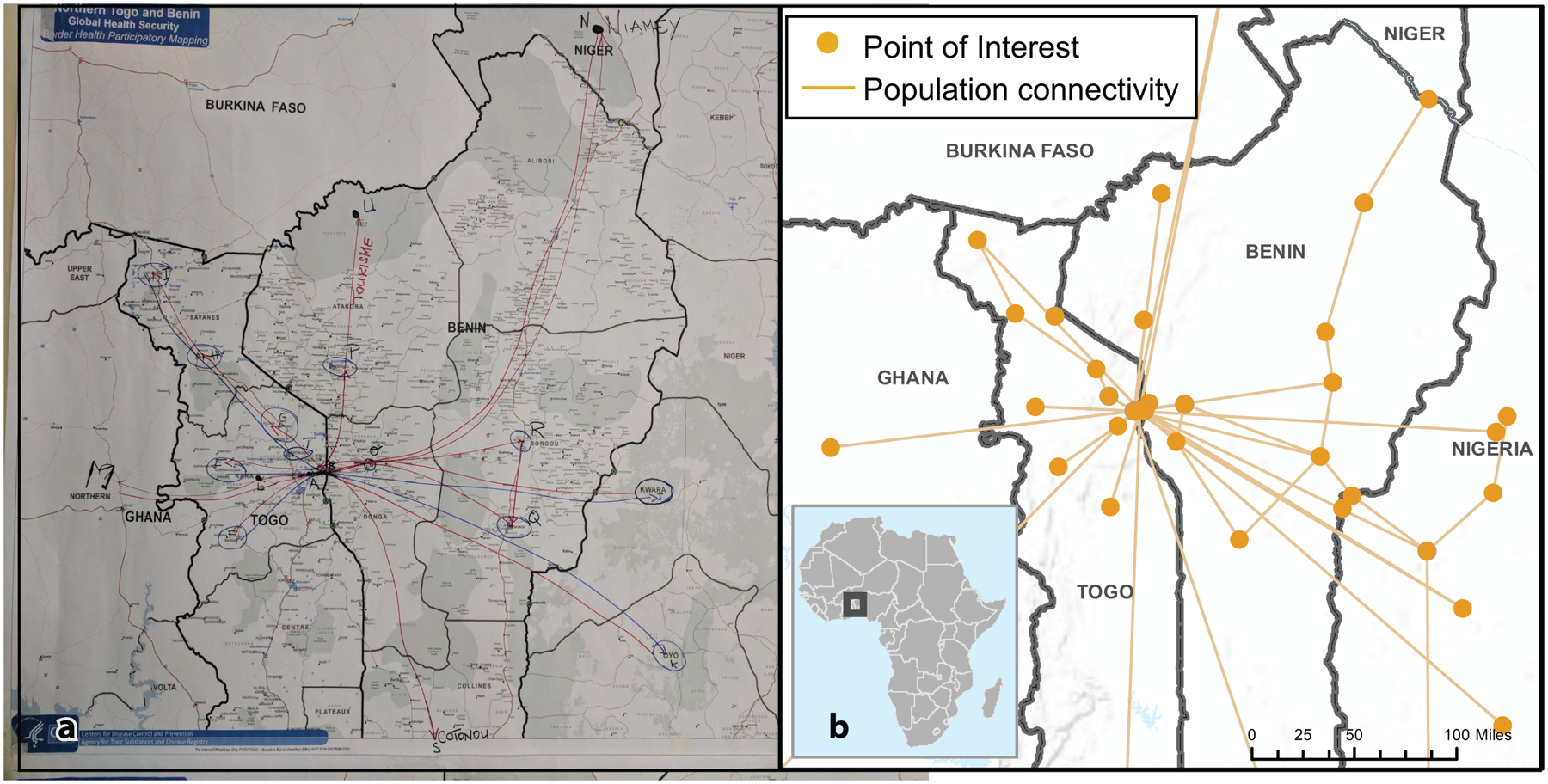
Results from a focus group discussion with border officials in Kémérida, Togo during Round 2 Population Connectivity Across Borders (PopCAB) implementation, April 2018. **a** Photo of an annotated map completed during the focus group discussion with border officials; **b** Digital summary of the annotations and additional spatial information captured only in the discussion notes.

Field team members participated in a 4-day, country-level workshop that included two days of training in a central location on the PopCAB method, one day of FGD implementation in the field teams’ assigned locations, and one day of debrief in the central location. The 4-day training and fieldwork workshop in Kara, Togo was followed immediately by a repeat 4-day workshop in Parakou, Benin resulting in 54 newly-trained MOH staff implementing 21 PopCAB FGD events across 14 cities within eight days (Fig. 4, Table 2). These FGDs included 224 community-level participants who consented to participate willingly and who represented the six stakeholder groups. During the debrief days, field teams presented an overview of their results and reflections on the training and field activity processes (Fig. 5). The team processed the qualitative and spatial data to create databases that captured 167 points of interest and 176 routes. They also determined that additional PopCAB events were not required to accomplish the defined objectives.

### Round 2, Phase 3.

**Fig. 6:**
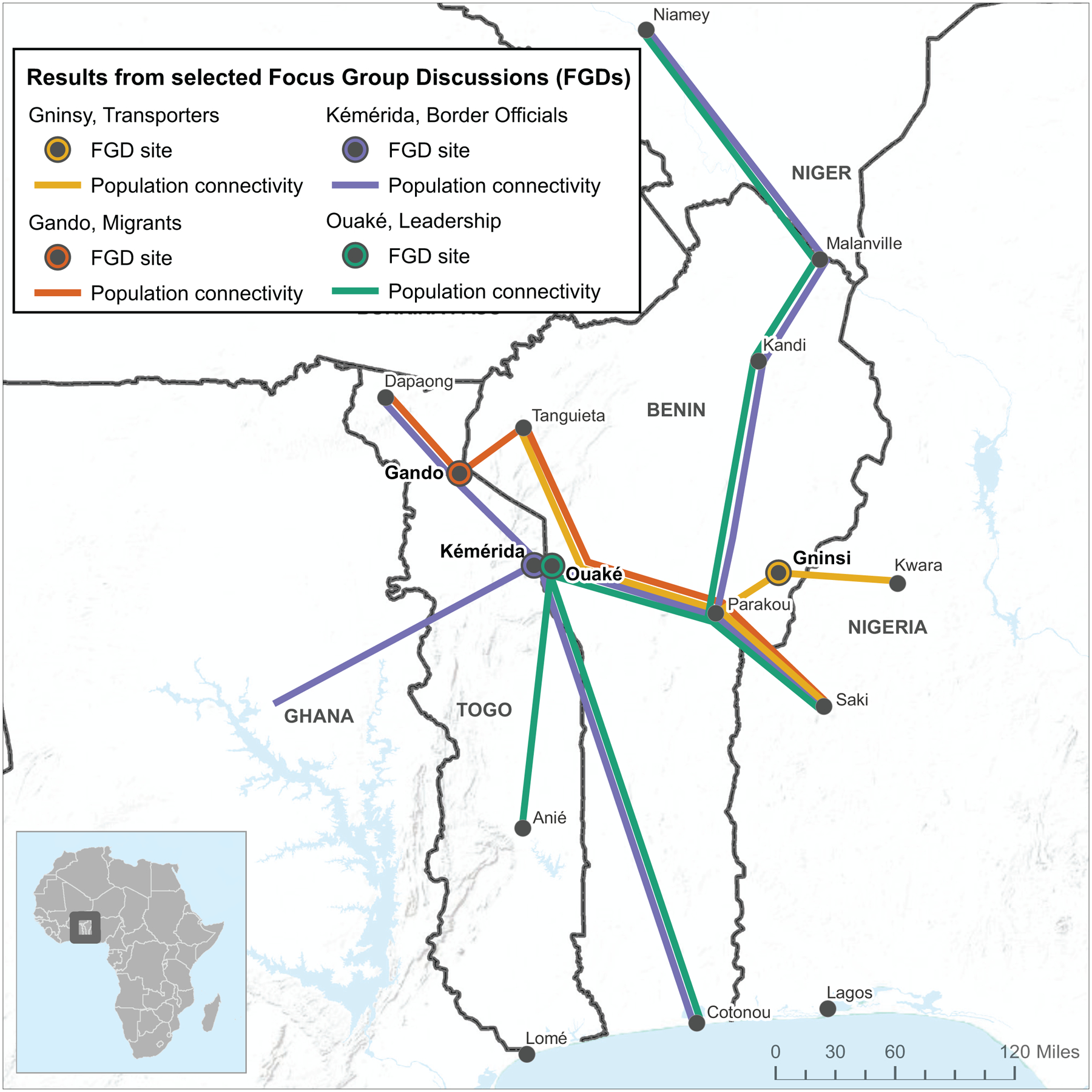
An example results map from Round 2 Population Connectivity Across Borders (PopCAB) implementation in Togo and Benin summarizing results from selected focus group discussions. Each color highlights a summary of locations or points of interest (black circles) and routes (lines) described by participants in each of the four focus group discussions. The location of each event is marked by a colored circle around a location.

The team created maps illustrating general trends in multinational migration pathways Togolese and Beninois follow to participate in seasonal agricultural contract work in fertile land in Western Nigeria and preferred medical facilities and traditional healers along the routes (Fig. 6). To create the results maps, the team aimed to create digital replicas of the participatory mapping results while incorporating additional spatial information included only in the qualitative data. By combining the FGD results through the digitization process, the team produced maps that layered information from various stakeholder groups and geographic areas. Of note, when stakeholders described the same geographic location in different ways, i.e., a market, a town, or a place to seek care by a traditional healer, all descriptions were retained and assigned to the same point on the map, using overlaid symbology. Further, because the GIS analyst geocoded each unique point of interest and travel route, the team could manipulate the spatial data to produce maps that addressed various MOH inquires. For example, the team produced a map to illustrate results across the 21 FGDs describing where people traveled from to visit traditional healers in western Benin. The MOH could use these tools to catalyze further community engagement and data collection during preparedness or response initiatives.

### Round 2, Phase 4.

The team applied the narrative and visual results to develop strategies to integrate better regional migration in national and binational preparedness and response strategies for Lassa fever, an overall objective identified in Phase 1. These strategies included allocating additional support for cross-border collaboration and relationship building in geographic areas with increased cross-border connectivity, clarifying the process to formally engage neighboring countries during an outbreak, selecting health facilities for enhanced training given their connectivity to the seasonal migration routes, and considering additional techniques to better integrate traditional healers in surveillance and community education strategies. Further, they refined the PopCAB toolkit after addressing field team feedback on training and implementation. The MOH integrated the final toolkit in national- and district-level preparedness plans as a method to complete more robust risk assessments for geographic areas and population groups with a higher risk of exposure during communicable disease outbreak responses.

Importantly, MOH leadership rapidly applied the results and concepts from this April 2018 initiative when a new Lassa fever case was identified in December 2018 in Borgou, Benin, along the border with Nigeria, in an individual who recently traveled into Benin from Nigeria. They rapidly identified the connection between the index case and the PopCAB results illuminating the sociocultural and medical and public health behaviors among a broader community with similar demographic and migration attributes. The Benin MOH immediately reached out to Togo MOH counterparts to enhance cross-border preparedness in targeted communities, followed the established procedure to inform national-level counterparts in Nigeria about the evolving context, and more rapidly and thoroughly gathered, documented, and visualized travel history data for the index and subsequent cases. These actions led to a more integrated multinational response.

## Discussion

Through PopCAB implementation in March 2017 and April 2018, Togo and Benin MOH, collaborating with ALCO and CDC, gathered and visualized multisectoral information on cross-border population movement through a participatory approach with multiple health and non-health, national- and community-level stakeholders. They used the results to design targeted binational preparedness and response strategies that enhanced cross-border surveillance and health education in communities and health care facilities with increased risk of disease importation and contact tracing efforts in distant, cross-border communities with elevated cultural connectivity to identified cases. The MOH expressed that without PopCAB, they would not have identified as rapidly or successfully the links between identified Lassa fever cases and the risk of where the outbreak could spread within the country and across borders. This sentiment was critical given the application in an environment with high cross-border movement (Dreier and Sow, 2015; International Centre for Migration Policy Development, 2015).

By implementing PopCAB, national, subregional, and local MOH staff were directly involved, for the first time, in community engagement efforts designed to gather qualitative and spatial data to guide public health resource allocation based on informal movement patterns. This effort led to national- and binational-level discussions about how to address the health context of mobile populations more effectively guided by a richer understanding of sociocultural connectivity across the region (Kakaī et al., 2020; Dangerfield, Vyska, and Gilligan, 2019; Airhihenbuwa et al., 2020).

The PopCAB method has limitations. Without clearly defined objectives and FGD or KII facilitation skills, the qualitative discussions can lead to gathering a little information about many locations and routes rather than detailed, relational information about a limited number of higher-priority locations and routes. Consequently, the team may not be able to guide strategic shifts in resource allocation strategies. Besides providing sufficient training, an implementation team can address this limitation by completing more thorough desk reviews of published information before implementing PopCAB events. Analyses and results interpretation are reliant on the distribution of included geographic areas and stakeholders. To appropriately interpret the results, the team should always present the summary information concurrently with where and with which stakeholder groups PopCAB data collection events occurred (Handcock and Gile, 2010). Notably, the team should emphasize that results do not reflect social network analyses and do not measure the strengthen of association between places or groups. Instead, by implementing the PopCAB toolkit, public health practitioners gather information about migration and population movement patterns for decision making in a way that can complement rigorous network analyses. Finally, to develop the robust spatial databases and results maps, the team must include a qualified GIS analyst with sufficient software to geocode the annotated and mentioned locations and routes.

Other MOH have used PopCAB for preparedness and response. The Uganda MOH implemented PopCAB during the 10th Ebola outbreak in the Democratic Republic of the Congo (DRC) to guide cross-border preparedness (Nakiire et al., 2020; Nanziri et al., 2020). More specifically, they used population movement information gathered through stakeholder participation not limited to border officials, transporters, health facility nurses, and fisherman to identify health facilities in Uganda with increased cross-border connectivity. In June 2019, one of these prioritized health facilities identified the first Ebola case in Uganda, in a child who had returned from travel to DRC (WHO 2019).

Togo and Benin MOH led a series of PopCAB initiatives during an outbreak response, as well as during preparedness efforts to illuminate social, behavioral, and cultural patterns of cross-border population movement through community engagement. The activities leveraged existing knowledge about social and cultural networks in the region, generated through complementary techniques such as social network analysis and mathematical modeling, to select priority geographic locations and stakeholders. Taken together, integrating PopCAB in public health practice to understand population movement patterns can help countries mitigate the international spread of disease.

## Figures and Tables

**Table 1.** General list of Population Connectivity Across Borders (PopCAB) questions included in key informant interview and focus group discussion guide template for national-level events

Please identify and describe, using the map, areas of high public health priority in *[insert country]*.
*The facilitator can select locations to discuss in more detail. As needed, engage the participants:* For all the priority areas we discussed, which are the most important? Please describe why these locations are the most important. *The facilitator can select locations to discuss in more detail. For the geographic areas of highest priority identified in question 1 and 2, ask the remaining questions*.
*WHO:* Describe the characteristics of travelers who travel into/through/out of this area. *WHY:* Describe why these people visit the area. *WHERE from:* Describe where people come from when they visit this area. Where do they go after they visit the area? *HOW:* Describe how people reach the area. *DURATION:* Describe how long to people stay when they visit this area.

**Table 2.** PopCAB focus group discussions completed in April 2018, Togo and Benin

Country	Location	Stakeholder
Benin	Anadana	Community Leadership
Benin	Boukoumbe	Community Leadership
Benin	Boukoumbe	Migrants
Benin	Gninsy	Transporter
Benin	Kabo	Community Leadership
Benin	Kassouala	Community Leadership and traditional healers
Benin	Ouaké	Community Leadership
Benin	Ouénou	Migrants
Benin	Parakou	Transporter / taxi driver
Benin	Tandou	Community Leadership
Benin	Tandou	Traditional healer
Benin	Tchikandou	Community Leadership
Togo	Gando	Community members
Togo	Gando	Migrants
Togo	Kémérida	Border officials
Togo	Kémérida	Community members
Togo	Kémérida	Traditional healer
Togo	Nadoba	Community Leadership
Togo	Nadoba	Migrants
Togo	Solla	Community Leadership
Togo	Solla	Migrants

## Data Availability

The datasets generated during or analyzed during this study are not publicly available because of potentially sensitive information about vulnerable populations. Still, the co-authors are processing the database to prepare it for public access. The data are available from the corresponding author on reasonable request.
